# The forgotten *p*-versine and *p*-coversine family of functions revisited

**DOI:** 10.1371/journal.pone.0308529

**Published:** 2024-08-13

**Authors:** Ali Hamzah Alibrahim, Saptarshi Das

**Affiliations:** 1 Centre for Environmental Mathematics, Faculty of Environment, Science and Economy, University of Exeter, Penryn, United Kingdom; 2 Mathematics Department, College of Science, Jouf University, Sakaka, Al Jouf, Saudi Arabia; 3 Institute for Data Science and Artificial Intelligence, University of Exeter, Exeter, Devon, United Kingdom; COMSATS University Islamabad, PAKISTAN

## Abstract

This study delves into special class of generalized *p*-trigonometric functions, examining their connection to the established counterparts like *p*-cosine and *p*-sine functions. We here explore the new class of functions called the *p*-versine, *p*-coversine, *p*-haversine, and *p*-hacovercosine, by providing comprehensive definitions and properties. Grounded in the characteristics of *p*-cosine and *p*-sine functions, the newly proposed functions offer unique mathematical insights. Our work contributes towards a thorough understanding of these new special functions, showcasing their potential applications in diverse scientific domains, from mathematical analysis to physics and engineering. This paper contributes as a valuable resource for future applied mathematics researchers, engaging with these new mathematical functions, enhancing the ability to model complex patterns from diverse real-world applications.

## 1 Introduction

The generalized complex numbers were introduced in [1, 2] as in the following:

$$C_{p}≔\left\{x+iy:x,y\in R;i^{2}=p;p\in R_{-}\right\}.$$

The *p*-trigonometric functions are generalizations of standard trigonometric functions sine, cosine, tangent and cotangent. For last few decades, the *p*-trigonometric functions have been extensively studied from various points of views by many researchers, particularly in the fields of signal processing and control systems [3, 4]. In $C_{p}$ the *p*-trigonometric functions *p*-cosine, *p*-sine, *p*-tangent and *p*-cotangent are defined as follows:

$$\begin{array}{c} \text{cosp}\left(\theta_{p}\right)=\text{cos}\;(\sqrt{\left|p\right|}.\theta_{p}), \end{array}$$ (1)

$$\begin{array}{c} \text{sinp}(\theta_{p})=\frac{1}{\sqrt{\left|p\right|}}\text{sin}\;(\sqrt{\left|p\right|}.\theta_{p}), \end{array}$$ (2)

$$\begin{array}{c} \text{tanp}(\theta_{p})=\frac{\text{sinp}(\theta_{p})}{\text{cosp}(\theta_{p})}, \end{array}$$ (3)

$$\begin{array}{c} \text{cotp}(\theta_{p})=\frac{\text{cosp}(\theta_{p})}{\text{sinp}(\theta_{p})}. \end{array}$$ (4)

In this paper, we introduce some new *p*-trigonometric functions which are generalizations of standard trigonometric functions viz. versine, coversine, haversine, and hacovercosine which were previously studed in [5–10].

## 2 The *p*-versine functions

**Definition 2.1**. For *p* < 0 the function *p*-versine, denoted by versinp is defined by:

$$\begin{array}{ccc} \text{versinp}(\theta_{p}) & = & 1-\text{cosp}(\theta_{p})\\ & = & 1-\text{cos}(\theta_{p}\;\sqrt{\left|p\right|}). \end{array}$$ (5)

We observe that versinp:R→[0,2].

**Remark 2.1**. (1) versinp is periodic function with period $\frac{2\pi }{\sqrt{\left|p\right|}}$.

(2) Differentiation formula:

$$\begin{array}{c} \frac{d}{d\theta_{p}}(\text{versinp}(\theta_{p}))=-p\;\text{sinp}\left(\theta_{p}\right)=\left|p\right|\;\text{sinp}\left(\theta_{p}\right). \end{array}$$ (6)

(3) The McLaurin series of versinp is given by:

$$\begin{array}{c} \text{versinp}\left(\theta_{p}\right)=-\sum_{n=1}^{\infty }\frac{p^{n}}{(2n)!}\;.\;\theta_{p}^{2n}. \end{array}$$ (7)

**Definition 2.2**. The function $\text{versinp}:[0,\frac{\pi }{\sqrt{\left|p\right|}}]\to \;\left[0,2\right]$ is bijective and its inverse ${\text{versinp}}^{-1}:\left[0\;,2\right]\to [0,\frac{\pi }{\sqrt{\left|p\right|}}]$.

**Remark 2.2**.

$$\begin{array}{c} {\text{versinp}}^{-1}\circ \;\text{versinp}\left(\theta_{p}\right)=\theta_{p}\;\;\forall \;\theta_{p}\;\in \;[0\;,\;\frac{\pi }{\sqrt{\left|p\right|}}]. \end{array}$$ (8)

$$\begin{array}{c} \text{versinp}\;\circ \;{\text{versinp}}^{-1}\left(\theta_{p}\right)=\theta_{p}\;\;\forall \;\theta_{p}\;\in \;\left[0,\;2\right]. \end{array}$$ (9)

**Remark 2.3**. (i) $\text{versinp}\left(0\right)=0\Rightarrow {\text{versinp}}^{-1}\left(0\right)=0$.

(ii) $\text{versinp}(\frac{\pi }{3\sqrt{\left|p\right|}})=\frac{1}{2}\Rightarrow \;{\text{versinp}}^{-1}(\frac{1}{2})=\frac{\pi }{3\sqrt{\left|p\right|}}$.

(iii) $\text{versinp}(\frac{\pi }{\sqrt{\left|p\right|}})=2\Rightarrow \;{\text{versinp}}^{-1}\left(2\right)=\frac{\pi }{\sqrt{\left|p\right|}}$.

**Theorem 2.3**. The function ${\text{versinp}}^{-1}:\left[0\;,2\right]\to [0\;,\frac{\pi }{\sqrt{\left|p\right|}}]$ is differentiable on (0, 2) and we have:

$$\begin{array}{c} \frac{d}{dx}({\text{versinp}}^{-1}\left(x\right))=\frac{1}{\sqrt{\left|p\right|}}\;.\frac{1}{\sqrt{x\;(2-x)}}. \end{array}$$ (10)

*Proof*.

$$\begin{array}{ccc} & & {\text{versinp}}^{-1}\circ \text{versinp}\left(x\right)=x\\ & \Rightarrow & \frac{d}{dx}({\text{versinp}}^{-1}\circ \text{versinp}\left(x\right))=1\\ & \Rightarrow & \frac{d}{dx}({\text{versinp}}^{-1}\left(\text{versinp}(x))\right)\;.\;\frac{d}{dx}(\text{versinp}(x))=1\\ & \Rightarrow & \frac{d}{dx}({\text{versinp}}^{-1}\left(\text{versinp}\left(x\right)\right))\;.\;\left|p\right|\;\text{sinp}\left(x\right)=1\\ & \Rightarrow & \frac{d}{dx}({\text{versinp}}^{-1}\left(\text{versinp}\left(x\right)\right))=\frac{1}{\left|p|\;\text{sinp}(x\right)}. \end{array}$$

On the other hand since

$${\text{cosp}}^{2}\left(x\right)-p{\;\text{sinp}}^{2}\left(x\right)=1\Rightarrow \left|p\right|\;{\text{sinp}}^{2}\left(x\right)=1-{\text{cosp}}^{2}\left(x\right).$$

Therefore we have:

$$\begin{array}{ccc} \sqrt{\left|p\right|}\;\text{sinp}\left(x\right) & = & \sqrt{1-{\text{cosp}}^{2}\left(x\right)}\\ & = & \sqrt{\text{versinp}(x)\;.\;(1+(1-\text{versinp}(x))}\\ & = & \sqrt{\text{versinp}(x)\;.\;(2-\text{versinp}(x))}. \end{array}$$

Hence,

$$\begin{array}{ccc} \frac{d}{dx}({\text{versinp}}^{-1}\left(\text{versinp}\left(x\right)\right)) & = & \frac{1}{\left|p|\;\text{sinp}(x\right)}\\ & = & \frac{1}{\sqrt{\left|p\right|}}\;.\;\frac{1}{\sqrt{|p|\;}\;\text{sinp}\left(x\right)}\\ & = & \frac{1}{\sqrt{\left|p\right|}}\;.\;\frac{1}{\sqrt{\text{versinp}(x)\;.\;(2-\text{versinp}(x))}}. \end{array}$$

Therefore,

$$\begin{array}{c} \frac{d}{dx}({\text{versinp}}^{-1}\left(x\right))=\;\frac{1}{\sqrt{\left|p\right|}}\;.\;\frac{1}{\sqrt{x\;.\;(2-x)}}. \end{array}$$

**Remark 2.4**. when *p* = −1 we get:

$$\begin{array}{c} \frac{d}{dx}({\text{vers}}^{-1}\left(x\right))=\frac{1}{\sqrt{x\;.\;(2-x)}}\;\forall \;x\in \left(0,2\right), \end{array}$$ (11)

see [5].

## 3 The *p*-coversine functions

We introduce the function *p*-coversine denoted by coversinp as follows:

$$\begin{array}{ccc} \text{coversinp}(x) & = & 1-\text{sinp}(x)\\ & = & 1-\frac{1}{\sqrt{\left|p\right|}}\;\text{sin}(\theta_{p}\sqrt{\left|p\right|})\\ & = & \frac{\sqrt{\left|p\right|}\;-\;\text{sin}\left(\theta_{p}\;\sqrt{\left|p\right|}\right)}{\sqrt{\left|p\right|}}. \end{array}$$ (12)

**Remark 3.1**. For this function, we have the following bounds:

$$\begin{array}{c} 1-\frac{1}{\sqrt{\left|p\right|}}\leq \text{coversinp}\left(\theta_{p}\right)\leq 1+\frac{1}{\sqrt{\left|p\right|}}. \end{array}$$ (13)

In fact,

$$\begin{array}{ccc} & & -1\leq -\text{sin}(\theta_{p}\;\sqrt{\left|p\right|})\leq 1\\ & \Rightarrow & \frac{\sqrt{\left|p\right|}-1}{\sqrt{\left|p\right|}}\leq \frac{\sqrt{\left|p\right|}-\text{sin}\left(\theta_{p}\;\sqrt{\left|p\right|}\right)}{\sqrt{\left|p\right|}}\leq \frac{1+\sqrt{\left|p\right|}}{\sqrt{\left|p\right|}}\\ & \Rightarrow & 1-\frac{1}{\sqrt{\left|p\right|}}\leq \text{coversinp}\left(\theta_{p}\right)\leq 1+\frac{1}{\sqrt{\left|p\right|}}. \end{array}$$

**Definition 3.1**. The function $\text{coversinp}:[-\frac{\pi }{2\sqrt{\left|p\right|}}\;,\frac{\pi }{2\sqrt{\left|p\right|}}]\to [1-\frac{1}{\sqrt{\left|p\right|}}\;,\;1+\frac{1}{\sqrt{\left|p\right|}}]$ is bijective and its inverse

$${\text{coversinp}}^{-1}:[1-\frac{1}{\sqrt{\left|p\right|}}\;,\;1+\frac{1}{\sqrt{\left|p\right|}}]\to [-\frac{\pi }{2\sqrt{\left|p\right|}}\;,\frac{\pi }{2\sqrt{\left|p\right|}}].$$

**Remark 3.2**. Connection with the inverse function:

$$\begin{array}{c} {\text{coversinp}}^{-1}\;\circ \text{coversinp}\left(\theta_{p}\right)=\theta_{p}\;\;\;\;\;\;\forall {\;\;\theta }_{p}\;\in [-\frac{\pi }{2\sqrt{\left|p\right|}}\;,\frac{\pi }{2\sqrt{\left|p\right|}}], \end{array}$$ (14)

$$\begin{array}{c} \text{coversinp}\;\circ {\text{coversinp}}^{-1}\left(\theta_{p}\right)=\theta_{p}\;\;\;\;\;\;\forall \;\;\theta_{p}\;\in [1-\frac{1}{\sqrt{\left|p\right|}}\;,\;1+\frac{1}{\sqrt{\left|p\right|}}]. \end{array}$$ (15)

**Theorem 3.2**. The function ${\text{coversinp}}^{-1}:[1-\frac{1}{\sqrt{\left|p\right|}}\;,\;1+\frac{1}{\sqrt{\left|p\right|}}]$ is differentiable on $\left(1-\frac{1}{\sqrt{\left|p\right|}}\;,\;1+\frac{1}{\sqrt{\left|p\right|}}\right)$ and

$$\begin{array}{c} \frac{d}{d\theta_{p}}({\text{coversinp}}^{-1}\left(\theta_{p}\right))=\frac{1}{\sqrt{\left(1-\sqrt{\left|p\right|}\;+\sqrt{\left|p\right|}\;\theta_{p}\right)\;.\;\left(1+\sqrt{\left|p\right|}-\sqrt{\left|p\right|}\;\theta_{p}\right)}}. \end{array}$$ (16)

*Proof*.

$$\begin{array}{c} {\text{coversinp}}^{-1}\;\circ \text{coversinp}\left(\theta_{p}\right)=\theta_{p}\\ \Rightarrow \frac{d}{d\theta_{p}}({\text{coversinp}}^{-1}\circ \text{coversinp}\left(\theta_{p}\right))=1\\ \Rightarrow \frac{d}{d\theta_{p}}({\text{coversinp}}^{-1}\left(\text{coversinp}\left(\theta_{p}\right)\right))=1 \end{array}$$

$$\begin{array}{c} \Rightarrow \frac{d}{d\theta_{p}}({\text{coversinp}}^{-1}\left(\text{coversinp}\left(\theta_{p}\right)\right))\;.\;\frac{d}{d\theta_{p}}(\text{coversinp}(\theta_{p}))=1\\ \Rightarrow \frac{d}{d\theta_{p}}({\text{coversinp}}^{-1}\left(\text{coversinp}\left(\theta_{p}\right)\right))\;.(-\text{cosp}(\theta_{p}))=1 \end{array}$$

$$\begin{array}{ccc} \Rightarrow \frac{d}{d\theta_{p}}({\text{coversinp}}^{-1}\left(\text{coversinp}\left(\theta_{p}\right)\right)) & = & \frac{\left(-1\right)}{\text{cosp}(\theta_{p})}\\ & = & \frac{\left(-1\right)}{\sqrt{1+p{\;\text{sinp}}^{2}\left(\theta_{p}\right)}} \end{array}$$

Now, using the following identity:

$$\begin{array}{c} \left(1+p{\;\text{sinp}}^{2}\left(\theta_{p}\right)\right)=\left(1+p{\left(1-\text{coversinp}\left(\theta_{p}\right)\right)}^{2}\right), \end{array}$$

we get:

$$\begin{array}{ccc} \frac{d}{d\theta_{p}}({\text{coversinp}}^{-1}\left(\text{coversinp}\left(\theta_{p}\right)\right)) & = & \frac{\left(-1\right)}{\sqrt{1-\sqrt{\left|p\right|}+\sqrt{\left|p\right|}\;\text{coversinp}\left(\theta_{p}\right)}}\\ & & .\frac{\left(-1\right)}{\sqrt{1+\sqrt{\left|p\right|}-\sqrt{\left|p\right|}\;\text{coversinp}\left(\theta_{p}\right)}}, \end{array}$$

$$\begin{array}{ccc} \frac{d}{d\theta_{p}}({\text{coversinp}}^{-1}\left(\theta_{p}\right)) & = & \frac{\left(-1\right)}{\sqrt{1-\sqrt{\left|p\right|}+\sqrt{\left|p\right|}\;\;\theta_{p}}}\;.\;\frac{\left(-1\right)}{\sqrt{1+\sqrt{\left|p\right|}-\sqrt{\left|p\right|}\;\;\theta_{p}}} \end{array}.$$

**Remark 3.3**. when *p* = −1 we get:

$$\begin{array}{c} \frac{d}{d\theta }({\text{coversin}}^{-1}\left(\theta \right))=\frac{\left(-1\right)}{\sqrt{\theta \;(2-\theta )}}, \end{array}$$ (17)

see [11].

**Fig 1:**
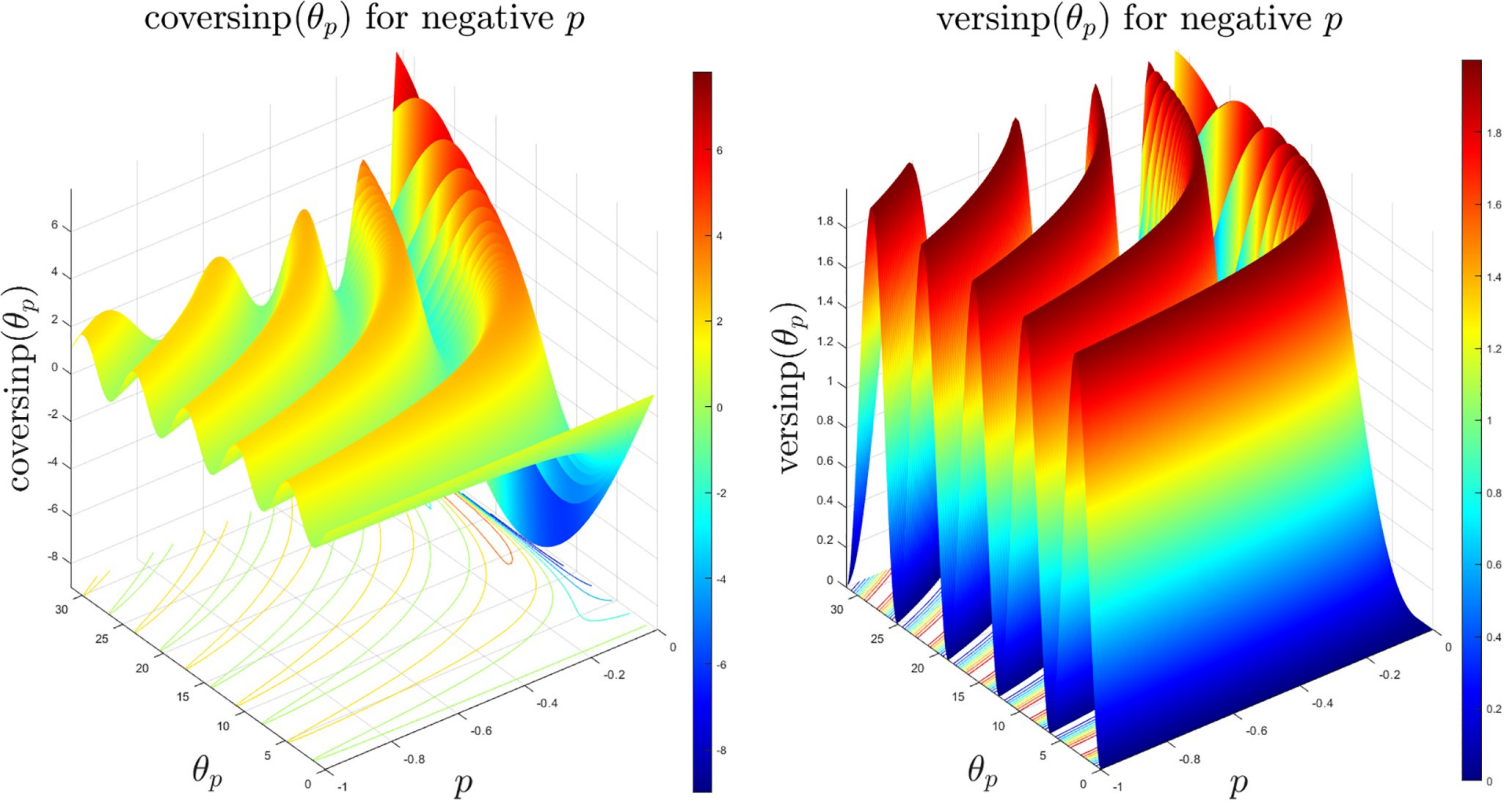
3D surface plots of the coversinp and versinp functions with *θ* ∈ [0, 10*π*] and *p* ∈ [−1, 0).

**Fig 2:**
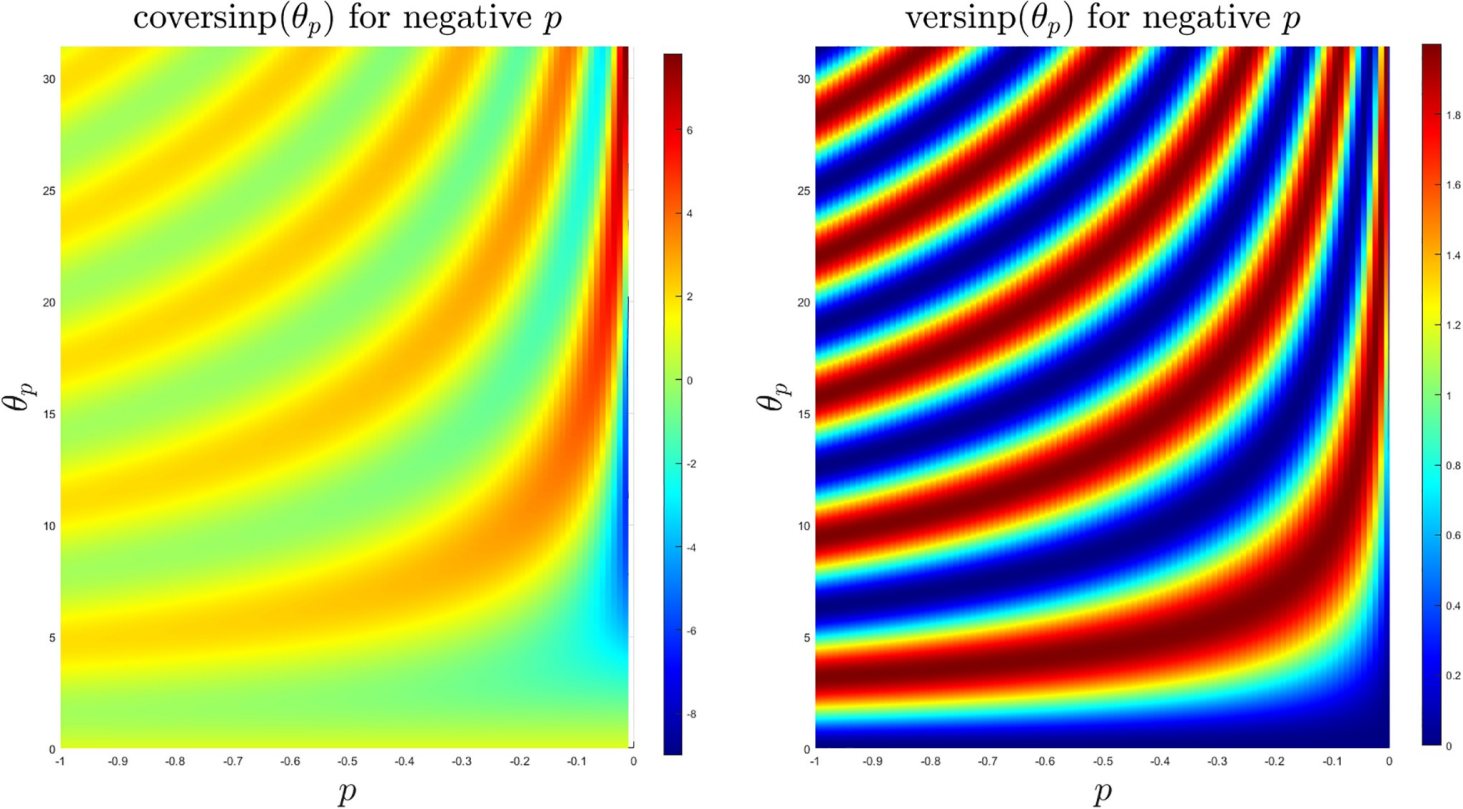
2D contour plots of the coversinp and versinp functions with *θ* ∈ [0, 10*π*] and *p* ∈ [−1, 0).

**Fig 3:**
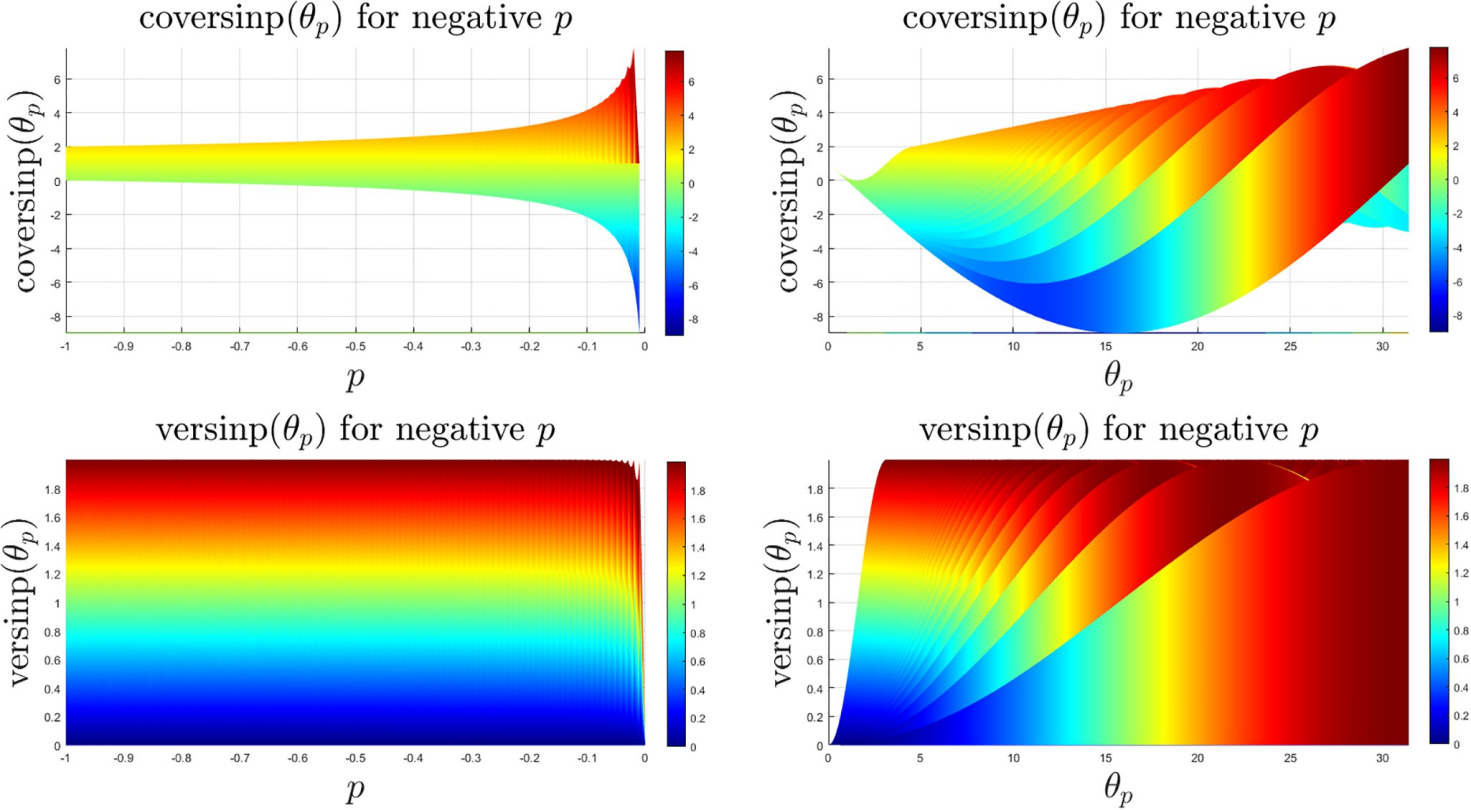
2D projections of the surface plots of the coversinp and versinp functions with *θ* ∈ [0, 10*π*] and *p* ∈ [−1, 0).

The coversinp and versinp family of functions for different values of *p* share some common characteristics since their structural form is similar. In Fig 1, the surface plots of these new classes of special functions are shown. It is evident that the versinp maintains the same peak while the maxima of the coversinp function gradually decreases with negative values of *p*. The corresponding surface plot between the angle *θ*_*p*_ and *p* are shown in Fig 2 where almost parallel contours are found for both coversinp and versinp family of functions. Next, we explore their variation with one of the parameters. This indicates that the peak of the coversinp function increases irregularly with higher values of *p* and *θ*_*p*_. Whereas, a smoother and periodic pattern is observed for the versinp family of functions as shown in the respective 2D projections in Fig 3.

## 4 The *p*-vercosine and *p*-covercosine functions

These two functions are given by:

$$\begin{array}{c} \text{vercosinp}(\theta )=1+\text{cosp}(\theta ), \end{array}$$ (18)

$$\begin{array}{c} \text{covercosinp}(\theta )=1+\text{sinp}(\theta ). \end{array}$$ (19)

**Fig 4:**
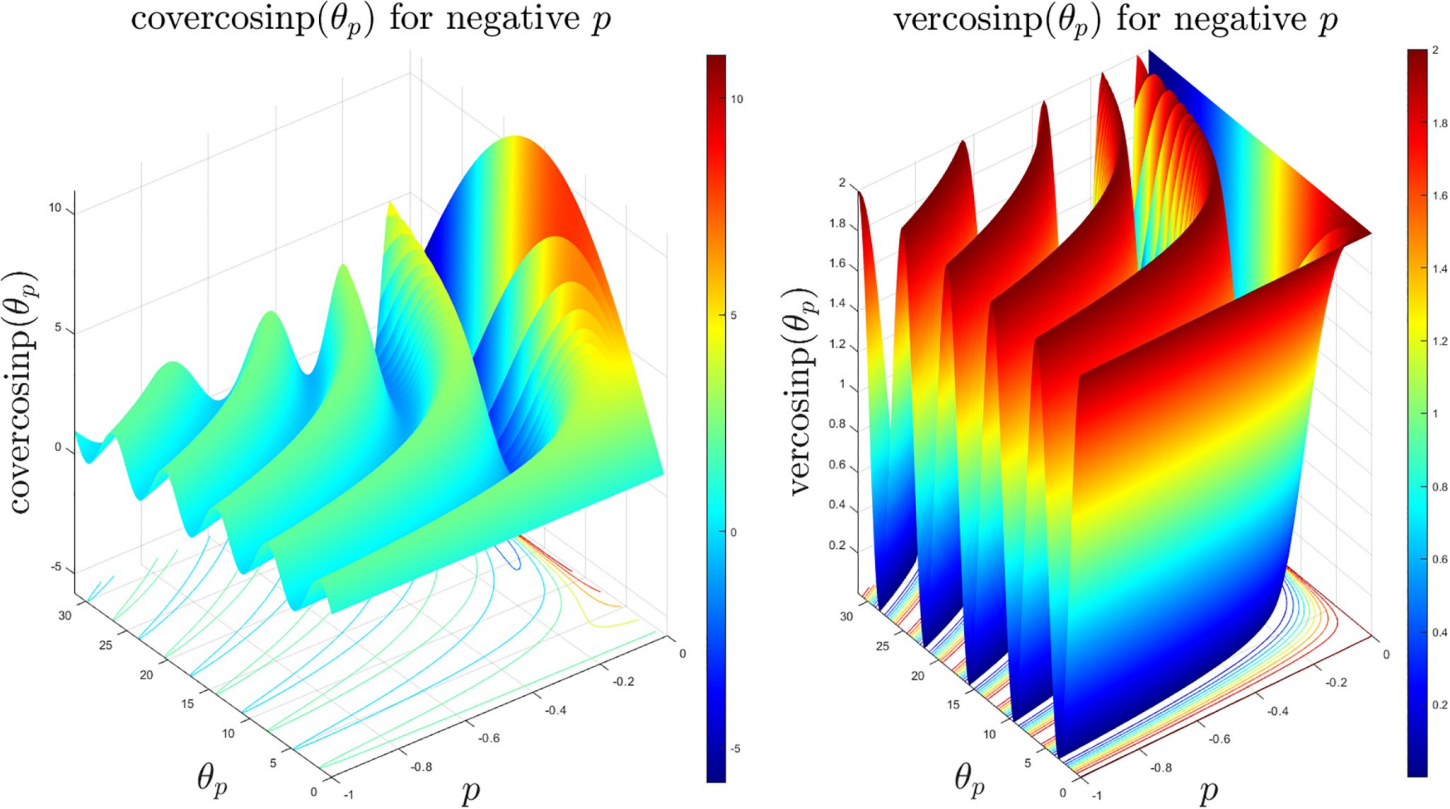
3D surface plots of the covercosinp and vercosinp functions with *θ* ∈ [0, 10*π*] and *p* ∈ [−1, 0).

**Fig 5:**
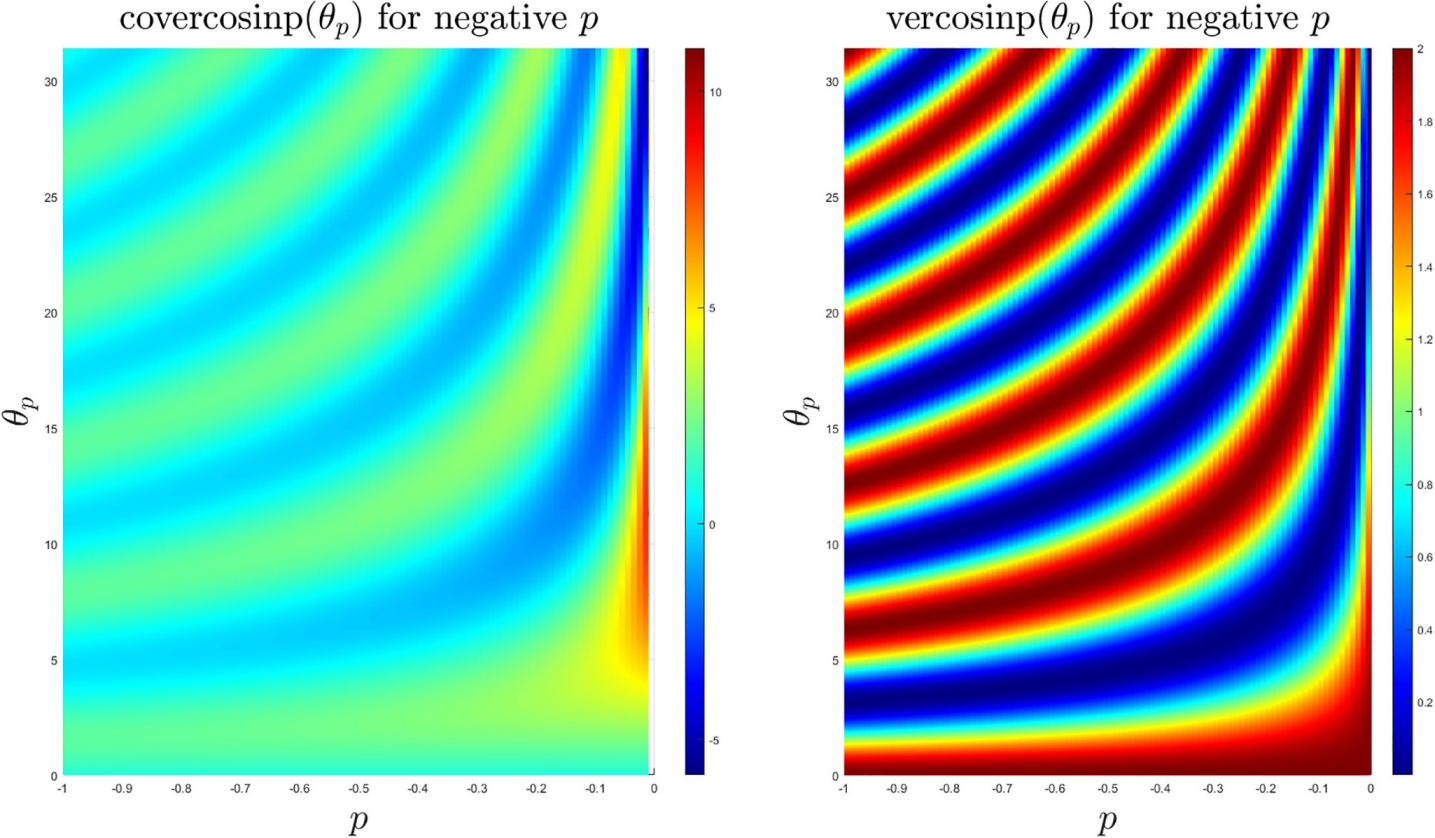
2D contour plots of the covercosinp and vercosinp functions with *θ* ∈ [0, 10*π*] and *p* ∈ [−1, 0).

**Fig 6:**
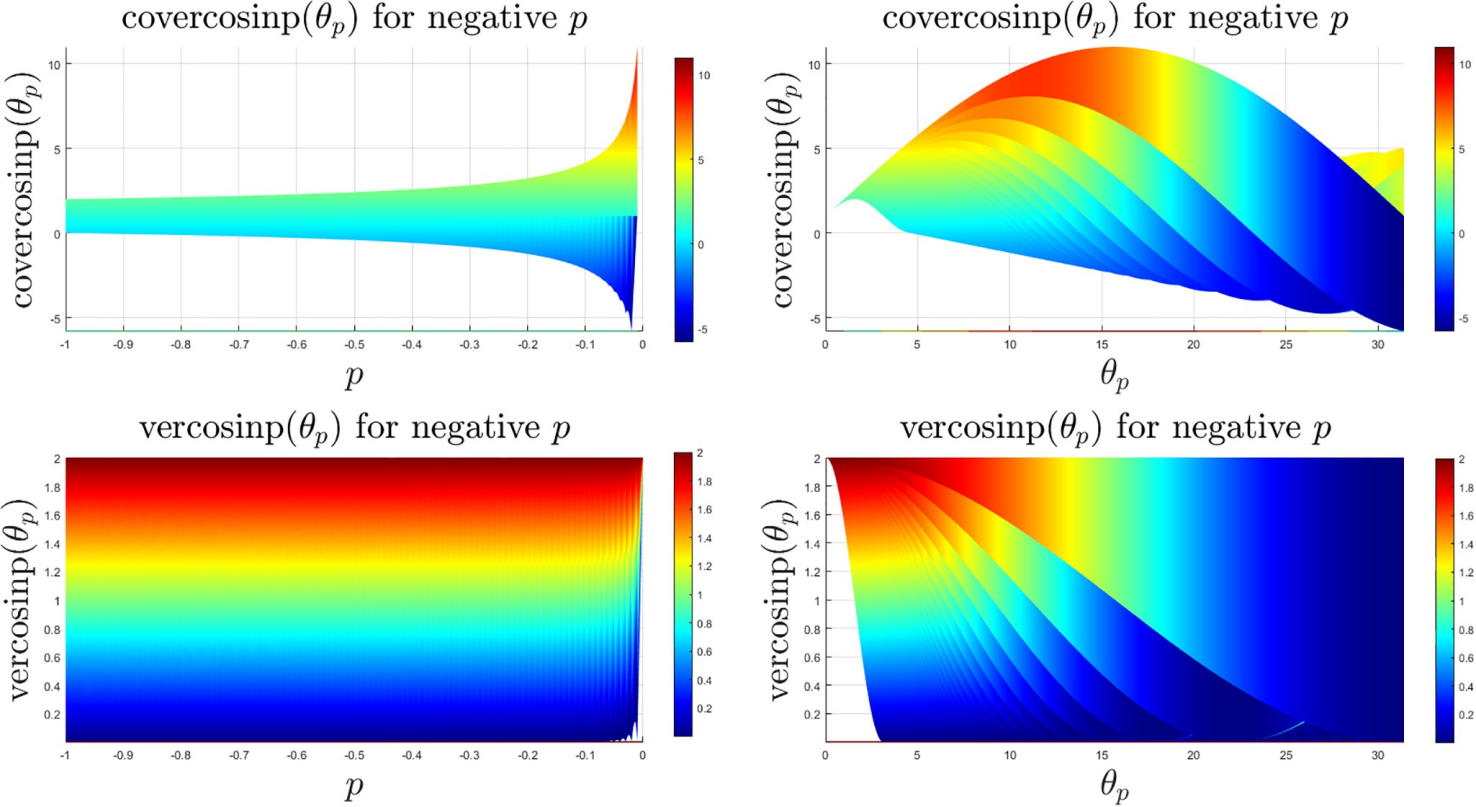
2D projections of the surface plots of the covercosinp and vercosinp functions with *θ* ∈ [0, 10*π*] and *p* ∈ [−1, 0).

They have similar shape like the sinp and cosp functions since these two functions represent just a scaled up version without changing the shape and due to the change in the sign of the cosp and sinp functions. However, due to change of sign before the cosp and sign functions, these vercosinp and covercosinp functions are a flipped version of the previously explored coversinp and versinp functions. The 3D surface plots are shown in Fig 4 which indicates that the peaks of the covercosinp keep increasing with higher *p* and *θ*_*p*_. Whereas a smoother periodic pattern is observed for the vercosinp family. The corresponding 2D contour plots in Fig 5 and the 2D projections in Fig 6 shows a scaled and flipped version of the functions, explored in the previous section.

## 5 The *p*-haversin and *p*-hacoversin functions

We then introduce the *p*-haversin function denoted by haversinp as follows:

$$\begin{array}{c} \text{haversinp}(\theta_{p})=\frac{1-\text{cosp}(\theta_{p})}{2|p|}. \end{array}$$ (20)

**Remark 5.1**. When *p* = −1, we get:

$$\begin{array}{c} \text{haversin}(\theta )=\frac{1-\text{cos}(\theta )}{2}, \end{array}$$ (21)

see [5].

**Lemma 5.1**. The haversinp function is bounded by:

$$\begin{array}{c} 0\leq \text{haversinp}\left(\theta_{p}\right)\leq \frac{1}{\left|p\right|}. \end{array}$$ (22)

*Proof*.

$$\begin{array}{ccc} -1\leq \text{cosp}(\theta_{p})\leq 1 & \Rightarrow & -1\leq -\text{cosp}(\theta_{p})\leq 1\\ & \Rightarrow & 0\leq 1-\text{cosp}(\theta_{p})\leq 2\\ & \Rightarrow & 0\leq \frac{1-\text{cosp}(\theta_{p})}{2\;|p|}\leq \frac{1}{\left|p\right|}\\ & \Rightarrow & 0\leq \text{haversinp}\left(\theta_{p}\right)\leq \frac{1}{\left|p\right|}. \end{array}$$

**Definition 5.2**. The function $\text{haversinp}:[0\;,\frac{\pi }{\sqrt{\left|p\right|}}]\to [0\;,\frac{1}{\left|p\right|}]$ is bijective and its inverse ${\text{haversinp}}^{-1}:\;[0\;,\;\frac{1}{\left|p\right|}]\to \;[0\;,\;\frac{\pi }{\sqrt{\left|p\right|}}]$.

**Remark 5.2**. Connection with the inverse function:

$$\begin{array}{c} {\text{haversinp}}^{-1}\circ \text{haversinp}\left(\theta_{p}\right)=\theta_{p}\;\;\;\;\forall \;\theta_{p}\;\in \;[0\;,\;\frac{\pi }{\sqrt{\left|p\right|}}], \end{array}$$ (23)

$$\begin{array}{c} \text{haversinp}\circ {\text{haversinp}}^{-1}\left(\theta_{p}\right)=\theta_{p}\;\;\;\forall \;\theta_{p}\;\in \;[0\;,\;\frac{1}{\left|p\right|}]. \end{array}$$ (24)

**Theorem 5.3**. The function ${\text{haversinp}}^{-1}:[0\;,\frac{1}{\left|p\right|}]\;\to [0\;,\frac{\pi }{\sqrt{\left|p\right|}}]$ is differentiable on $\left(0\;,\frac{1}{\left|p\right|}\right)$ and its derivative is given by:

$$\begin{array}{c} \frac{d}{d\theta_{p}}({\text{haversinp}}^{-1}\left(\theta_{p}\right))=\frac{1}{\sqrt{\left|p\right|}\;\sqrt{\theta_{p}\;(\frac{1}{\left|p\right|}\;-\theta_{p})}}. \end{array}$$ (25)

*Proof*.

$$\begin{array}{c} {\text{haversinp}}^{-1}\circ \text{haversinp}\left(\theta_{p}\right)=\theta_{p}\\ \Rightarrow \frac{d}{d\theta_{p}}({\text{haversinp}}^{-1}\circ \text{haversinp}\left(\theta_{p}\right))=1\\ \Rightarrow \frac{d}{d\theta_{p}}({\text{haversinp}}^{-1}\left(\text{haversinp}\left(\theta_{p}\right)\right))=1\; \end{array}$$

$$\begin{array}{c} \Rightarrow \frac{d}{d\theta_{p}}({\text{haversinp}}^{-1}\left(\text{haversinp}\left(\theta_{p}\right)\right))\;.\;\frac{d}{d\theta_{p}}(\text{haversinp}(\theta_{p}))=1\; \end{array}$$

$$\begin{array}{c} \Rightarrow \frac{d}{d\theta_{p}}({\text{haversinp}}^{-1}\left(\text{haversinp}\left(\theta_{p}\right)\right))=\frac{2}{\text{sinp}(\theta_{p})}=\frac{2\;\sqrt{\left|p\right|}}{\sqrt{1-{\text{cosp}}^{2}\left(\theta_{p}\right)}}. \end{array}$$

Now, using the following identity:

$$\begin{array}{ccc} 1-{\text{cosp}}^{2}\left(\theta_{p}\right) & = & \left(1-\text{cosp}\left(\theta_{p}\right)\right)\;.\;(1+\text{cosp}\left(\theta_{p}\right)\\ & = & (\;2\;|p|\;\text{haversinp}\left(\theta_{p}\right))\;.\;(2\;-2\;|p|\;\text{haversinp}\left(\theta_{p}\right))\\ & = & (4\;|p|\;\text{haversinp}\left(\theta_{p}\right))\;.\;(1-|p|\;\text{haversinp}\left(\theta_{p}\right)), \end{array}$$

we get:

$$\begin{array}{c} \frac{d}{d\theta_{p}}({\text{haversinp}}^{-1}\left(\text{haversinp}\left(\theta_{p}\right)\right))=\frac{2\;\sqrt{\left|p\right|}}{2\;\sqrt{|p|\;\text{haversinp}\left(\theta_{p}\right)\;.\;(1-|p|\;\text{haversinp}\left(\theta_{p}\right)}}, \end{array}$$

$$\begin{array}{c} \frac{d}{d\theta_{p}}({\text{haversinp}}^{-1}\left(\theta_{p}\right))=\frac{1}{\sqrt{|p|\;}\;\;\sqrt{\theta_{p}\;\;(\frac{1}{\left|p\right|}-\theta_{p})}} \end{array}.$$

**Remark 5.3**. When *p* = −1, we get:

$$\begin{array}{c} \frac{d}{d\theta }({\text{haversin}}^{-1}\left(\theta \right))=\frac{1}{\;\;\sqrt{\;\theta \;(1-\theta )}}. \end{array}$$ (26)

A similar treatment can be adopted for the hacoversin functions which has the following form:

$$\begin{array}{c} \text{hacoversinp}(\theta )=\frac{1-\text{sinp}(\theta )}{2}. \end{array}$$ (27)

For brevity, similar derivations for hacoversin are omitted.

## 6 The *p*-havercosin and *p*-hacovercosin functions

We introduce the two functions as follows:

$$\begin{array}{c} \text{havercosinp}\left(\theta_{p}\right)=\frac{1+\text{cosp}(\theta_{p})}{2}, \end{array}$$ (28)

$$\begin{array}{c} \text{hacovercosinp}\left(\theta_{p}\right)=\frac{1+\text{sinp}(\theta_{p})}{2\;|p|}. \end{array}$$ (29)

In rest of this section, we show derivation of few properties only for one of these two functions due to their similarity in the structure relating to the fundamental sinp and cosp special functions.

**Remark 6.1**. When *p* = −1, identity (29) coincides with

$$\begin{array}{c} \text{hacovercosin}\left(\theta \right)=\frac{1+\text{sin}(\theta )}{2}. \end{array}$$ (30)

**Lemma 6.1**. For *p* < 0, the following inequalities hold:

$$\begin{array}{c} \frac{1}{2|p|}(1-\frac{1}{\sqrt{\left|p\right|}})\leq \text{hacovercosinp}\left(\theta_{p}\right)\leq \frac{1}{2\;|p|}(1+\frac{1}{\sqrt{\left|p\right|}}). \end{array}$$ (31)

*Proof*.

$$\begin{array}{ccc} & & \frac{-1}{\sqrt{\left|p\right|}}+1\leq 1+\text{sinp}\left(\theta_{p}\right)\leq \frac{1}{\sqrt{\left|p\right|}}+1\;\\ & \Rightarrow & \frac{1}{2|p|}(1-\frac{1}{\sqrt{\left|p\right|}})\leq \frac{1+\text{sinp}(\theta_{p})}{2|p|}\leq \frac{1}{2|p|}(1+\frac{1}{\sqrt{\left|p\right|}})\\ & \Rightarrow & \frac{1}{2|p|}(1-\frac{1}{\sqrt{\left|p\right|}})\leq \text{hacovercosinp}\left(\theta_{p}\right)\leq \frac{1}{2\;|p|}(1+\frac{1}{\sqrt{\left|p\right|}}). \end{array}$$

**Definition 6.2**. The function:

$$\text{hacovercosinp}:[\frac{-\pi }{2\sqrt{\left|p\right|}}\;,\frac{\pi }{2\sqrt{\left|p\right|}}]\to [\frac{1}{2\;|p|}(1-\frac{1}{\sqrt{\left|p\right|}}),\frac{1}{2\;|p|}(1+\frac{1}{\sqrt{\left|p\right|}})]$$

is bijective and its inverse

$${\text{hacovercosinp}}^{-1}:[\frac{1}{2\;|p|}(1-\frac{1}{\sqrt{\left|p\right|}}),\frac{1}{2\;|p|}(1+\frac{1}{\sqrt{\left|p\right|}})]\to [\frac{-\pi }{2\sqrt{\left|p\right|}}\;,\frac{\pi }{2\sqrt{\left|p\right|}}].$$

**Remark 6.2**. Connection with the inverse function:

$$\begin{array}{c} {\text{hacovercosinp}}^{-1}\circ \text{hacovercosinp}\left(\theta_{p}\right)=\theta_{p}\;\;\forall \;\theta_{p}\;\;\in \;[\frac{-\pi }{2\sqrt{\left|p\right|}}\;,\frac{\pi }{2\sqrt{\left|p\right|}}], \end{array}$$ (32)

$$\begin{array}{c} \text{hacovercosinp}\circ {\text{hacovercosinp}}^{-1}\left(\theta_{p}\right)=\theta_{p}\;\forall \;\theta_{p}\in [\frac{1}{2\;|p|}(1-\frac{1}{\sqrt{\left|p\right|}})\;,\frac{1}{2\;|p|}(1+\frac{1}{\sqrt{\left|p\right|}})]. \end{array}$$ (33)

**Theorem 6.3**. The function

$${\text{hacovercosinp}}^{-1}:[\frac{1}{2\;|p|}(1-\frac{1}{\sqrt{\left|p\right|}}),\frac{1}{2\;|p|}(1+\frac{1}{\sqrt{\left|p\right|}})]\to [\frac{-\pi }{2\sqrt{\left|p\right|}}\;,\frac{\pi }{2\sqrt{\left|p\right|}}]$$

is differentiable on $\left(\frac{1}{2\;|p|}(1-\frac{1}{\sqrt{\left|p\right|}})\;,\frac{1}{2\;|p|}(1+\frac{1}{\sqrt{\left|p\right|}})\right)$ and

$$\begin{array}{c} \frac{d}{d\theta_{p}}({\text{hacovercosinp}}^{-1}\left(\theta_{p}\right))=\frac{2\;|p|}{\sqrt{1+p\;(2\;|p|\;\theta_{p}{-1)}^{2}}}. \end{array}$$ (34)

*Proof*. From the identity (32) we may write:

$$\begin{array}{c} \frac{d}{d\theta_{p}}({\text{hacovercosinp}}^{-1}\circ \text{hacovercosinp}\left(\theta_{p}\right))=1\; \end{array}$$

$$\begin{array}{c} \Rightarrow \frac{d}{d\theta_{p}}({\text{hacovercosinp}}^{-1}\left(\text{hacovercosinp}\left(\theta_{p}\right)\right))\;.\frac{d}{d\theta_{p}}(\text{hacovercosinp}(\theta_{p}))=1 \end{array}$$

$$\begin{array}{c} \Rightarrow \frac{d}{d\theta_{p}}({\text{hacovercosinp}}^{-1}(\text{hacovercosinp}(\theta_{p}))).\frac{\text{cosp}(\theta_{p})}{2|p|}=1 \end{array}$$

$$\begin{array}{c} \Rightarrow \frac{d}{d\theta_{p}}({\text{hacovercosinp}}^{-1}(\text{hacovercosinp}(\theta_{p})))=\frac{2\;|p|}{\text{cosp}(\theta_{p})} \end{array}$$

$$\begin{array}{c} \Rightarrow \frac{d}{d\theta_{p}}({\text{hacovercosinp}}^{-1}(\text{hacovercosinp}(\theta_{p})))=\frac{2|p|}{\sqrt{1+p\;{\text{sinp}}^{2}(\theta_{p})}} \end{array}$$

$$\begin{array}{c} \Rightarrow \frac{d}{d\theta_{p}}({\text{hacovercosinp}}^{-1}(\text{hacovercosinp}(\theta_{p})))=\frac{2|p|}{\sqrt{1+p\;(2\;|p|\;\text{hacovercosinp}\left(\theta_{p}\right){-1)}^{2}}} \end{array}$$

$$\begin{array}{c} \Rightarrow \frac{d}{d\theta_{p}}({\text{hacovercosinp}}^{-1}\left(\theta_{p}\right))=\frac{2|p|}{\sqrt{1+p\;(2\;|p|\;\;\theta_{p}{-1)}^{2}}}. \end{array}$$

**Remark 6.3**. When *p* = −1, we get:

$$\begin{array}{c} \frac{d}{d\theta }({\text{hacovercosin}}^{-1}\left(\theta \right))=\frac{2}{\sqrt{1-{\left(2\theta -1\right)}^{2}}},\; \end{array}$$ (35)

see [9].

**Remark 6.4**. By observing that

$$\begin{array}{c} {\text{hacovercosinp}}^{-1}\left(\theta_{p}\right)={\text{sinp}}^{-1}(2\;|p|\;\theta_{p}-1)\; \end{array}$$ (36)

we can find:

$$\begin{array}{ccc} \frac{d}{d\theta_{p}}({\text{hacovercosinp}}^{-1}\left(\theta_{p}\right)) & = & \frac{d}{d\theta_{p}}({\text{sinp}}^{-1}(2\;|p|\;\theta_{p}-1))\\ & = & \frac{1}{\sqrt{1+p\;(2\;|p|\;\;\theta_{p}{-1)}^{2}}}\;.\;2\;\left|p\right|\\ & = & \frac{2|p|}{\sqrt{1+p\;(2\;|p|\;\;\theta_{p}{-1)}^{2}}}. \end{array}$$

**Fig 7:**
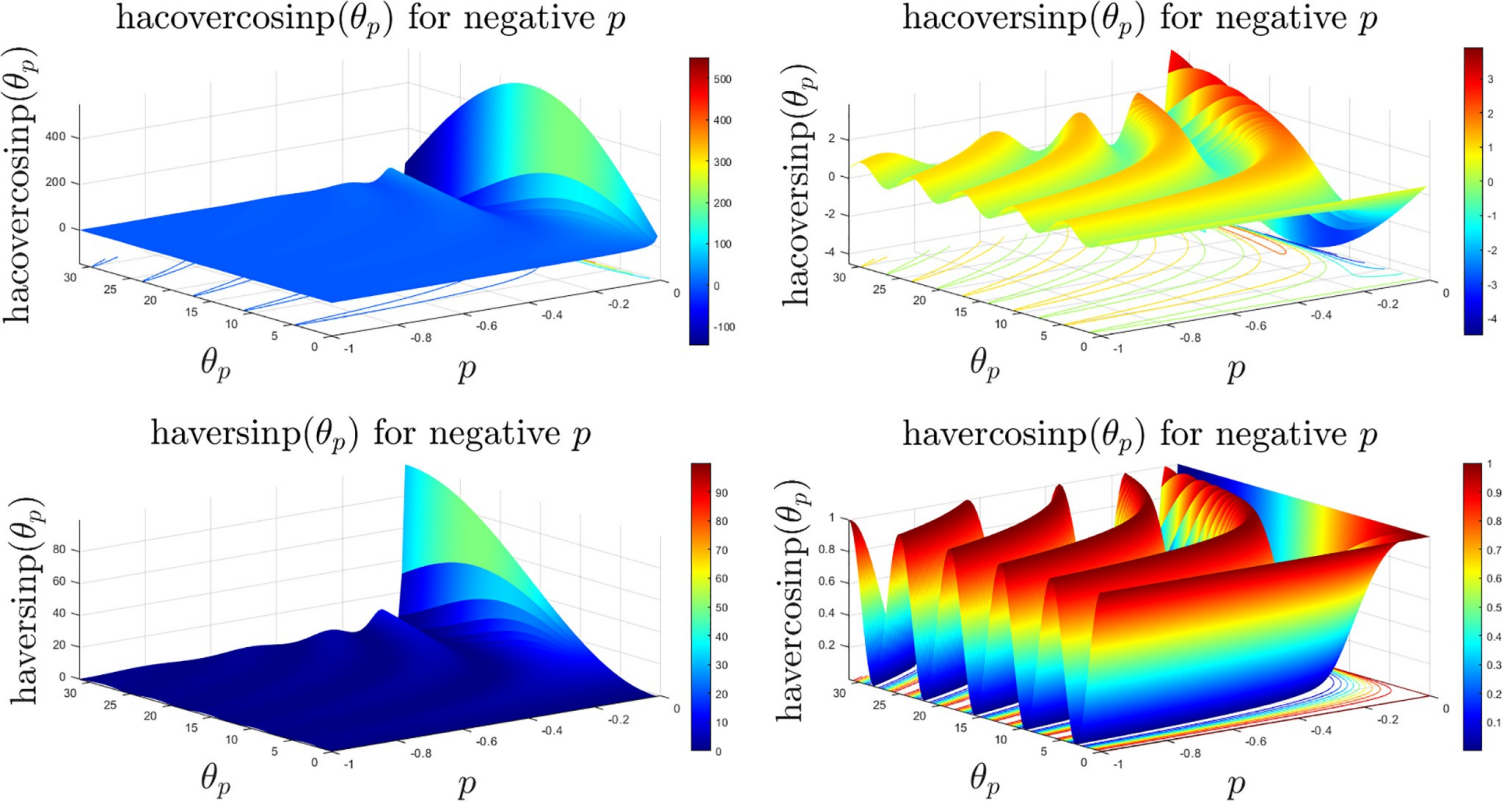
3D surface plots of the hacovercosinp, hacoversinp, haversinp and havercosinp functions with *θ* ∈ [0, 10*π*] and *p* ∈ [−1, 0).

The generic nature of these eight classes of new generalised *p*-special trigonometric functions are similar with a scaled and/or flipped version due to change of sign of the sinp and cosp special functions. The 3D suface plots of the hacovercosinp, hacoversinp, haversinp and havercosinp classes of special functions are shown in Fig 7. It is evident that the hacoversinp and havercosinp shares some degree of similarity with the function explored in earlier sections. However, hacovercosinp and haversinp functions are highly irregular and rapidly increases for higher *p* and *θ*_*p*_.

## 7 Nature of special functions and their patterns

**Fig 8:**
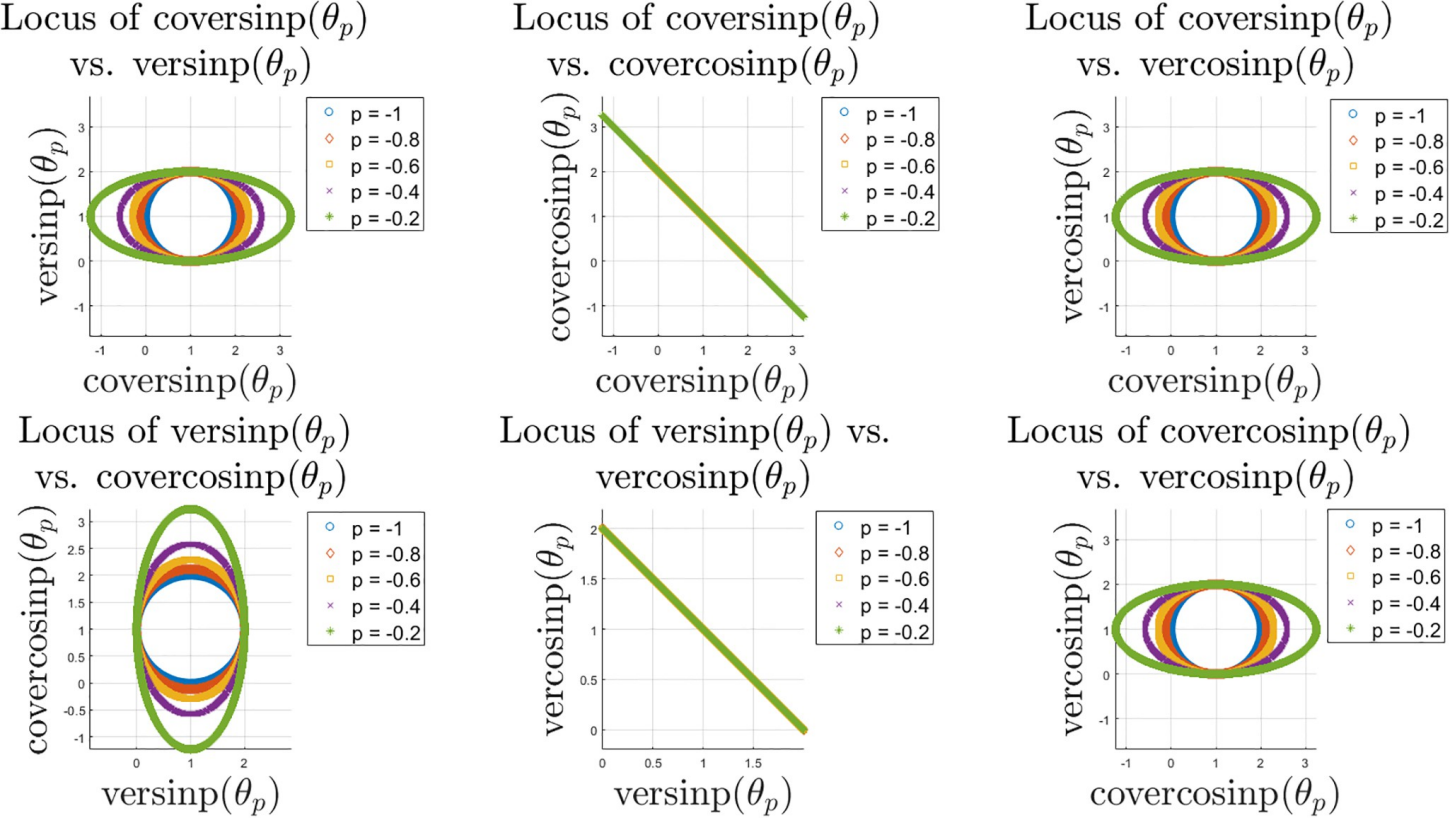
Locus of the ver and cover family of functions for *θ* ∈ [0, 10*π*] and *p* ∈ [−1, 0).

As an extension of the sinp and cosp classes of special functions, these new classes of trigonometric functions can help modelling interesting patterns in complex datasets, as opposed to algebraic functions. The complexity of the surface plots of these eight classes of new special functions are shown in the previous sections, whereas here we explore the scatter diagrams between different pairs of these classes of functions. First we show the locus of the ver and cover family of functions for different values of *p* in Fig 8. It appears that the eccentricity of the elliptical pattern increases with smaller *p* while the elliptical pattern may be stretched horizontally or vertically from a perfect circle, depending on the choice of function pairs.

**Fig 9:**
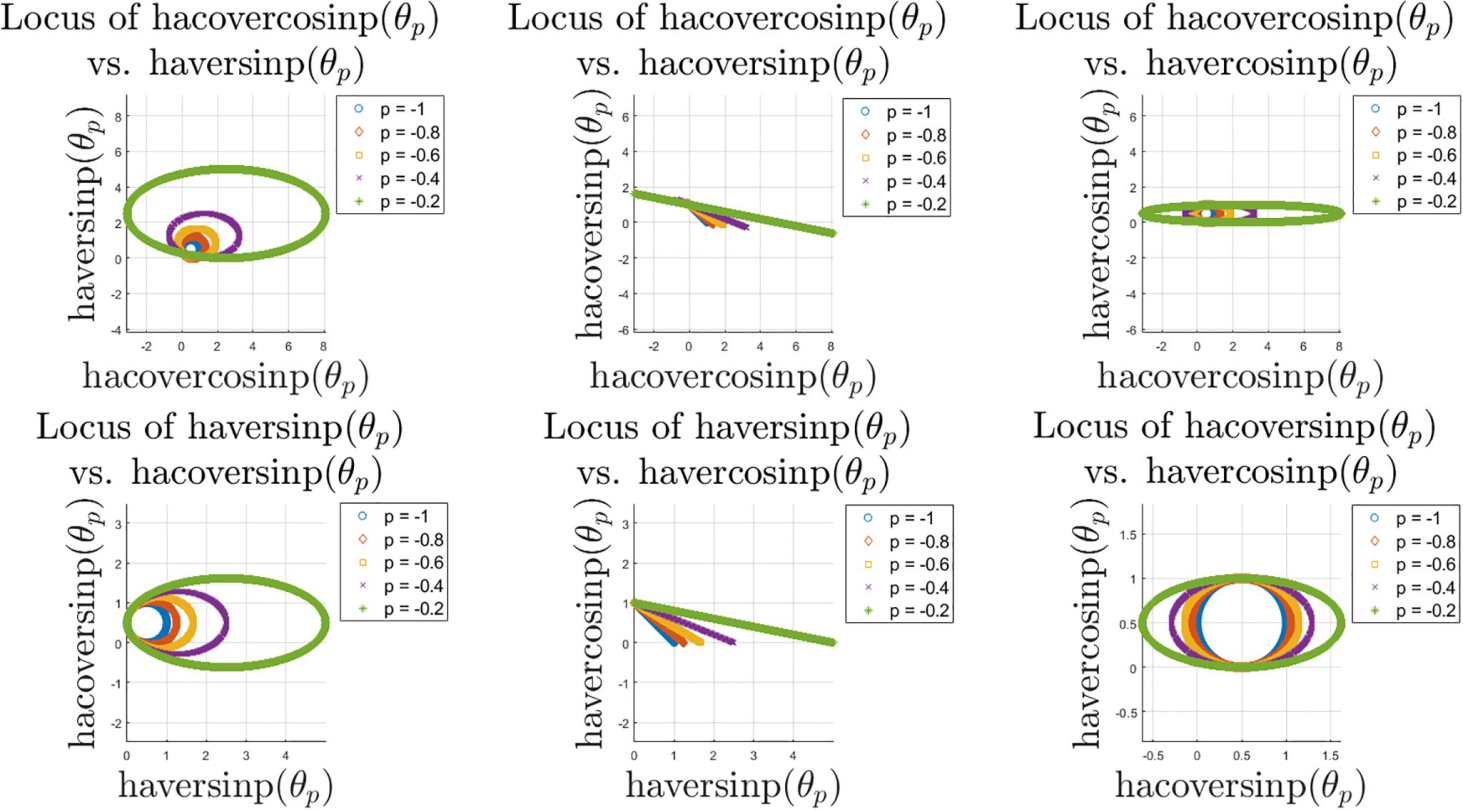
Locus of the haver and hacover family of functions for *θ* ∈ [0, 10*π*] and *p* ∈ [−1, 0).

**Fig 10:**
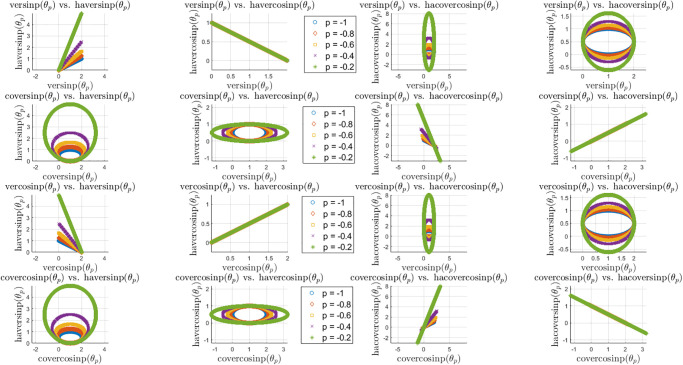
Locus of the ver/cover vs. haver/hacover family of functions for *θ* ∈ [0, 10*π*] and *p* ∈ [−1, 0).

Next, we show similar locus plots between the haver and hacover family of functions in Fig 9. Depending on the choice of function pairs, the elliptical patterns are stretched horizontally. However, as opposed to the ver and cover family, here the haver and hacover family of functions do not always maintain the same centroid except few cases. Some of the function pairs lead to linear relationships with negative slope due to the change of sign in the basic construct of these functions. Next, we explore the locus plots or patterns between ver/cover vs. haver/hacover family of special functions in Fig 10. These cases include linear relations with positive and negative slope, elliptical patterns with the same and also different centroids and eccentricities. The patterns involving the inverse of these functions are more involved and have not been explored hree for brevity.

## 8 Conclusion and future works

In this paper, we show the properties and characteristics of eight new classes of special trigonometric functions viz. *p*-versine, *p*-coversine, *p*-vercosine, *p*-covercosine, *p*-haversine, *p*-hacoversine, *p*-havercosine, and *p*-hacovercosine. Their definitions, general properties, rules, and uses are also shown. These special functions have a vast scope of usage in diverse scientific and engineering applications. Future scope of work may include statistical pattern modelling using these new classes of special functions [12], as compared to the classical trigonometric functions and algebraic functions, proving new identities involving them, and solving differential equations involving these new functions.

We have derived many important properties of these eight new classes of special functions like their domain of definition, differentiation rules, and connection with their inverse functions. This is the first paper of its kind to demonstrate the basic properties of these new classes of special functions. The scope of this paper does not allow to demonstrate immediate applications since the current contributions are in finding new mathematical functions. However, to encourage future researchers, there can be several real-world applications using these special functions. This may include use of these special functions to solve applied mathematics problems like ordinary and partial differential equations [13, 14], integral transforms [15] e.g. Laplace and Fourier analysis and complex analysis bridging the duality between the trigonometric and hyperbolic families. In terms of physical sciences and engineering applications, similar to the well-known trigonometric functions, these new special function can be used in signal and image processing using diverse filtering algorithms, stability and control of linear dynamical systems using transfer function models and their frequency domain analysis including root locus, Nyquist, Nichols and Bode plots, nonlinear dynamical systems [16], electrical engineering problems often using complex numbers like alternating current (AC) circuit analysis, phasor and power systems [17, 18]; also quantum mechanics, wave functions, electromagnetic fields, fluid dynamics, vibration analysis in mechanical, structural and civil engineering, channel modelling and modulation techniques in communication and electronics engineering, navigation and kinematics in aerospace and robotics, optical engineering and geophysics—literally all scientific disciplines that use complex numbers and trigonometric functions as their basic building blocks.
